# Pure additive contribution of genetic variants to a risk prediction model using propensity score matching: application to type 2 diabetes

**DOI:** 10.5808/GI.2019.17.4.e47

**Published:** 2019-12-23

**Authors:** Chanwoo Park, Nan Jiang, Taesung Park

**Affiliations:** 1 Department of Statistics, Seoul National University, Seoul 08826, Korea; 2 Interdisciplinary Program in Bioinformatics, Seoul National University, Seoul 08826, Korea

**Keywords:** genome-wide association study, penalized regression model, propensity score, type 2 diabetes

## Abstract

The achievements of genome-wide association studies have suggested ways to predict diseases, such as type 2 diabetes (T2D), using single-nucleotide polymorphisms (SNPs). Most T2D risk prediction models have used SNPs in combination with demographic variables. However, it is difficult to evaluate the pure additive contribution of genetic variants to classically used demographic models. Since prediction models include some heritable traits, such as body mass index, the contribution of SNPs using unmatched case-control samples may be underestimated. In this article, we propose a method that uses propensity score matching to avoid underestimation by matching case and control samples, thereby determining the pure additive contribution of SNPs. To illustrate the proposed propensity score matching method, we used SNP data from the Korea Association Resources project and reported SNPs from the genome-wide association study catalog. We selected various SNP sets via stepwise logistic regression (SLR), least absolute shrinkage and selection operator (LASSO), and the elastic-net (EN) algorithm. Using these SNP sets, we made predictions using SLR, LASSO, and EN as logistic regression modeling techniques. The accuracy of the predictions was compared in terms of area under the receiver operating characteristic curve (AUC). The contribution of SNPs to T2D was evaluated by the difference in the AUC between models using only demographic variables and models that included the SNPs. The largest difference among our models showed that the AUC of the model using genetic variants with demographic variables could be 0.107 higher than that of the corresponding model using only demographic variables.

## Introduction

Genome-wide association studies (GWASs) have identified many disease-related genetic variants, including numerous single-nucleotide polymorphisms (SNPs). Kooperberg et al. [1] constructed prediction models with SNPs and showed that they improved diagnosis and disease risk prediction. Bae et al. [2] constructed prediction models of quantitative traits using common genetic variants and compared several variable selection methods, including stepwise linear regression (SLR), least absolute shrinkage and selection operator (LASSO), and the elastic-net (EN) algorithm via mean square error. More recently, Bae et al. [3] compared several variable selection methods for predicting the risk of type 2 diabetes (T2D).

Some problems need to be considered when predicting disease risk according to genetic variants, and technologies are available that can help to solve these problems. First, the construction of prediction models suffers from the ‘large p, small n’ problem. That is, the number of genetic variants is much larger than the number of samples, which induces the curse of dimensionality [4]. Next, the presence of linkage disequilibrium, which refers to the non-random association of alleles in different loci, has impeded statistical inferences due to multi-collinearity [5,6]. Multi-collinearity makes parameter estimates non-stable and increases the estimates’ variance [7]. Third, only a small proportion of heritability has been explained by the SNPs discovered until now. This ‘missing heritability’ of complex diseases sometimes hinders the detection of SNPs with effects on complex diseases [8]. Many of the aforementioned problems have been an obstacle to disease risk prediction via genetic variants. Nonetheless, penalized regression has solved the ‘large p, small n’ problem, and missing heritability may be explained by newly identified SNPs, including rare variants.

Heritability is estimated as the ratio of variance caused by genetic factors to the total phenotypic variance [9]. Instead of heritability, in this study, we focus on the accuracy of prediction models. It should be noted that most of these prediction models have used SNPs, which represent genetic effects, and demographic variables, which represent environmental effects. However, it is not easy to evaluate the pure additive contribution of genetic variants in classically used demographic models. Since prediction models for T2D include some heritable traits, such as body mass index (BMI), the contribution of SNPs to T2D prediction using unmatched case-control samples may be underestimated [10,11]. In this article, we propose a method that uses propensity score matching (PSM) to determine the pure additive contribution of SNPs [12]. PSM helps avoid underestimating the contribution of the effects of genetic variants. It can also reduce possible confounding effects from demographic variables when unmatched samples are used. Thus, applying PSM enables the contribution of the effects of genetic variants to be more correctly estimated in a prediction model.

For an illustrative example of our approach, we selected T2D as a trait of interest. T2D results from the interactions between environmental factors and genetic factors. Many studies have sought to predict T2D through genetic variants [10,11,13,14]. Obesity is the strongest predictor of T2D, and several additional risk factors such as age, sex, smoking, and family history have been well identified [15-19]. Furthermore, some studies have shown that T2D is strongly associated with genetic factors [20]. Monozygotic twins had a T2D incidence matching rate of around 70%, whereas dizygotic twins had a T2D incidence matching rate of 20%–30% [21]. However, some skeptical opinions have been raised regarding arguments that SNPs are effective for predicting T2D. Lyssenko and Laakso [13] reviewed 43 different studies, and acknowledged that genetic variants create opportunities to improve the accuracy of T2D risk prediction, but pointed out that so far studies have not given compelling evidence to support the use of genetic variants for predicting T2D. Furthermore, Wray et al. [22] discussed some limitations and pitfalls of prediction analysis for complex traits and asserted that naïve assessments can lead to severe bias.

Some previous studies on T2D have been conducted using data from the Korea Association Resources (KARE) project [2,14]. However, previous studies have some deficiencies. First, prediction performance—assessed by testing area under the receiver operating characteristic curve (AUC) values—was overestimated due to overfitting. When selecting SNPs, previous studies used all training and test data together. The training data were then used to build prediction models. This way of selecting SNPs tends to yield higher test AUCs than expected. Second, although SNPs have an influence on traits, the inclusion of some heritable demographic variables in a prediction model may lead to an underestimation of the pure additive genetic contribution of SNPs.

In this study, we built prediction models for T2D following the methods proposed by Bae and colleagues [2,14], while performing valid SNP selection to avoid overfitting. We also investigated the pure additive contribution of SNPs to T2D prediction by comparing the performance of a prediction model with only demographic variables to that of a model with both SNPs and demographic variables [2]. We used data from the KARE project. To reduce possible confounding effects from demographic variables caused by using unmatched samples, we used PSM, which allowed us to create pairs constituting one case and one control with similar demographic variables. We used PSM to investigate the pure additive contribution of SNPs on T2D diagnosis and to avoid the effects of confounding.

We created three different SNP sets using combinations of variants from the GWAS catalog and statistically significant variants in Koreans [23]. We then used SLR, LASSO, and the EN algorithm for variable selection. Next, we created prediction models using logistic regression techniques such as SLR, LASSO, and EN. Finally, we calculated the AUC and compared the models that used only demographic variables with those that used demographic variables and genetic variants. For the LASSO-LASSO combination, which showed the largest difference among our models, it was found that the test AUC of the model that used genetic variants and demographic variables was 0.107 higher than that of the model using only demographic variables.

## Methods

### Korea Association Resource Project

The KARE project began in 2007 with Ansung and Ansan regional cohorts representative of the general Korean population. The Affymetrix Genome-Wide Human SNP array 5.0 (Affymetrix Inc., Santa Clara, CA, USA) was used to analyze the genotype data from 10,038 participants. After quality control with a Hardy-Weinberg equilibrium p-value < 10^-6^ and genotype call rates less than 95%, and with the exclusion of SNPs with a minor allele frequency < 0.05, a total of 305,799 autosomal SNPs were utilized in this analysis. After eliminating participants with samples having low call rates (less than 96%), contaminated samples, gender inconsistency, serious concomitant illness, and cryptic relatedness, 8,842 samples (4,183 males and 4,659 females) were included in the analysis. Since our study focused on T2D, we selected only T2D patients and controls by excluding 3,863 samples using the T2D diagnostic criteria summarized in Table 1 [24]. Table 2 presents the demographic information of participants and differences in demographic variables between cases and controls.

**Fig. 1.**
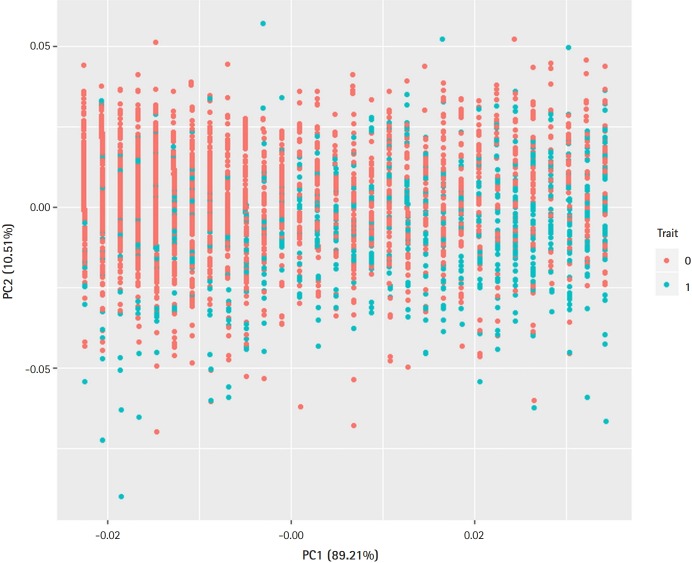
Principle component analysis plot. Demographic variables (sex, age, body mass index) discriminate the type 2 diabetes (T2D) cases from controls. Trait 0 (red), control; Trait 1 (blue), T2D.

Fig. 1 presents a principal component analysis plot that demonstrates the relationship between T2D and demographic variables. As can be seen in Fig. 1, demographic variation did not discriminate cases and controls well.

### Statistical analysis

SNPs were selected by two different approaches: from a single-SNP analysis and from the GWAS catalog [25]. Then, we built prediction models using logistic regression via SLR, LASSO, and EN.

### Propensity score matching

PSM is a statistical matching technique that attempts to estimate the effectiveness of treatments, policies, or other interventions by taking covariates into account [12]. PSM reduces the bias due to confounding variables. The propensity score is calculated by the following conditional probability. p(χi)=pr (T=1X=χi)=exp(γ0+γ1sexi+γ2agei+γ3BMIi)exp(γ0+γ1sexi+γ2agei+γ3BMIi)+1

The caliper is defined by the maximum propensity score difference within the matched pair. Three methods of matching individuals with similar propensity scores are presented based on the concept of the caliper in the R package *MatchIt*: largest, smallest, and random [26]. The ‘largest’ method establishes matches from the largest to the smallest value of a distance measure, while the ‘smallest’ method generates matches from the smallest to the largest value of a distance measure, while the ‘random’ method yields matches in random order. PSM was applied to the KARE data to ensure homogeneity of demographic variables (covariates) between the control and T2D groups, using the R package *MatchIt*.

Since it was necessary to minimize the loss of data due to the non-matched sample and the homogenization of covariates between controls and cases, we manipulated the caliper (from 0 to 1) by increments of 0.01. We checked the p-values using the paired t-test and the Wilcoxon test to evaluate the homogeneity of the cases’ and controls’ propensity scores at each caliper increment and for each method of choosing the caliper. For each caliper, we conducted 100 experiments. To ensure demographic homogeneity of the case and control group, we only considered calipers for which the p-values of both the paired t-test and the Wilcoxon test were larger than 0.05.

### SNP sets

As the GWAS catalog is based on populations of worldwide ancestry, while the KARE dataset is drawn from the Korean population, we carefully constructed three different SNP sets, which we denoted as KARE, GWAS + KARE, and CATAGENE. First, the KARE set consisted of the SNPs chosen by the p-values from a single-SNP analysis with adjustments for sex, age, and BMI. Second, the GWAS + KARE set was a combination of SNPs from the GWAS catalog (May 22, 2019) related to T2D and SNPs from the KARE data analysis. Third, the CATAGENE set was assembled through the steps detailed below. We first selected the genes in the GWAS catalog, and then extracted all SNPs in those genes from the KARE data. After performing a single-SNP analysis, we assembled the CATAGENE set based on the p-values. The SNPs were selected by the p-values of the univariate logistic regression for each SNP. The top 200, 500, and 1,000 SNPs were chosen based on these p-values for the prediction model.

We used only genotyped variants when choosing the candidate SNPs and constructing the prediction models. Therefore, non-genotyped variants were not included in our data, even if they were in the GWAS catalog. We found 132 SNPs in the GWAS catalog [25], and 11,025 catalog-related genes (SNPs located in the gene in which the GWAS catalog SNPs were located). Table 3 provides more details and further clarification on the SNP sets.

### Variable selection

At first, we randomly selected two-thirds of the samples for the training set, and the remaining third was used for the test set. Table 4 shows the sample size of the training set and test set, respectively. With the SNP sets we constructed earlier, the variable selection was conducted by SLR, LASSO, and EN to select SNPs via five-fold cross validation (CV) of the training set.

The penalized SLR model used the following formula: $log\frac{\pi_{i}}{1-\pi_{i}}=\beta_{0}+\beta_{1}x_{i1}+\beta_{2}x_{i2}+\cdots +\beta_{p}x_{ip}+\gamma_{1}sex_{i}+\gamma_{2}age_{i}+\gamma_{3}BMI_{i}$

In this formula, *π_i_* is the probability of T2D (1 ≤ *i* ≤ *n*), *n* denotes the number of samples. *x_ij_* represents the SNPs (1 ≤ *i* ≤ *n*, 1 ≤ *j* ≤ *p*) with 0, 1, and 2 values for the number of minor alleles. *p* denotes the number of SNPs used in the model. Stepwise selection was used to maximize the AUC by updating variables step by step. Since age, BMI, and sex are known demographic and prognostic variables of T2D, we fixed these three variables during the stepwise process. This procedure was performed using the R package *MASS* [27].

The LASSO and EN estimates of β were obtained by minimizing the following formula. $\sum_{i=1}^{n}{\left(y_{i}-\pi_{i}\right)}^{2}+\lambda_{1}\overset{n}{\underset{i=1}{\sum }}\left|\beta_{i}\right|\;\mathrm{for}\;\mathrm{LASSO},\;\mathrm{and}\;\sum_{i=1}^{n}{\left(y_{i}-\pi_{i}\right)}^{2}+\lambda_{1}\overset{n}{\underset{i=1}{\sum }}\left|\beta_{i}\right|+\lambda_{2}\sum_{i=1}^{n}\beta_{i}^{2}\;\mathrm{for}\;\mathrm{EN}$ $\mathrm{where}\;\pi_{i}:\;=\;\frac{1}{1+exp(-\beta^{T}\chi_{i}+\gamma_{1}sex_{i}+\gamma_{2}age_{i}+\gamma_{3}BMI_{i}))}$

Values of the parameter *λ* were estimated by CV, using the R package *glmnet* [28].

The following five groups were then defined:

(1)Group 1: SNPs that appeared at least once in the five-fold CV.

(2)Group 2: SNPs that appeared at least twice in the five-fold CV.

(3)Group 3: SNPs that appeared at least three times in the five-fold CV.

(4)Group 4: SNPs that appeared at least four times in the five-fold CV.

(5)Group 5: SNPs that appeared in every time in the five-fold CV.

These groups represent the sets of candidate SNPs selected by SLR, LASSO and EN, which were used to construct the prediction model.

### Prediction models

To make prediction models, we used the same prediction methods (logistic SLR, EN, and LASSO) that were used for variable selection. More specifically, for LASSO, we selected the λ value to be *lambda.min*, which is the value at which the training mean square error is smallest [28]. For EN, we selected the *λ* value to be *lambda.1se* in the *glmnet* package. Each prediction model was evaluated in terms of the test-set AUC.

## Results

### Propensity score matching

**Fig. 2.**
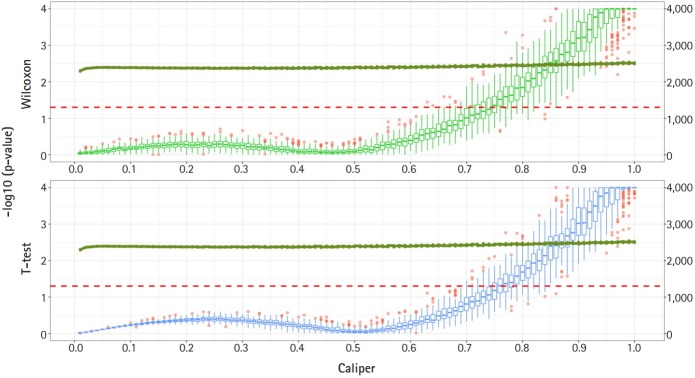
Propensity score matching results (matching method = “smallest”). Green boxes represent the p-values of the Wilcoxon test. Blue boxes mean the p-values of the paired t-test. The solid green lines represent the number of matched samples with the caliper as the x-axis. The red line means p = 0.05. The p-values are represented by a log scale.

**Fig. 3.**
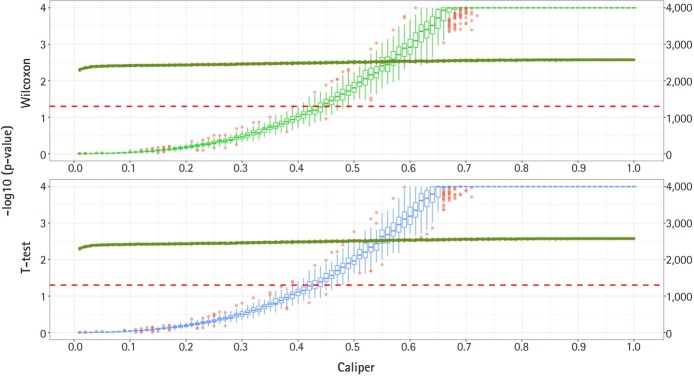
Propensity score matching results (matching method = “random”). Green boxes represent the p-values of the Wilcoxon test. Blue boxes mean the p-values of the paired t-test. The solid green lines represent the number of matched samples with the caliper as the x-axis. The red line means p = 0.05. The p-values are represented by a log scale.

**Fig. 4.**
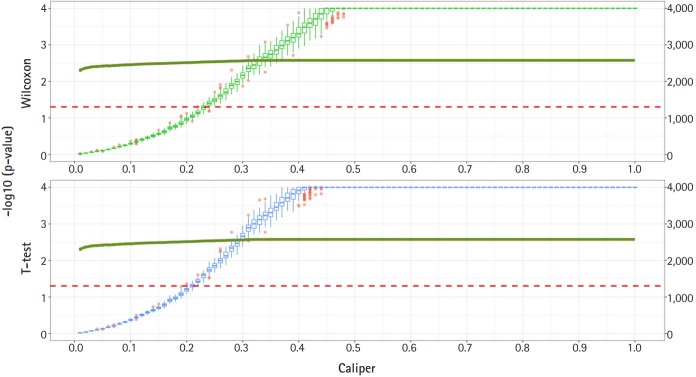
Propensity score matching results (matching method = “Largest”). Green boxes represent the p-values of the Wilcoxon test. Blue boxes mean the p-values of the paired t-test. The solid green lines represent the number of matched samples with the caliper as the x-axis. The red line means p = 0.05. The p-values are represented by a log scale.

Fig. 2 shows a graph of the relationship between the caliper and the p-values of the Wilcoxon test and t-test. Each box plot in the graph shows the confidence level of the p-values for the Wilcoxon test and the t-test. The right x-axis and green line show the average number of matched samples. Figs. 2–4 present the results of the various caliper selection methods (‘smallest,’ ‘largest,’ and ‘random’).

As described above, we conducted 100 experiments for each caliper. First, we selected the largest caliper for which the maximum value of the experiment’s p-value was > 0.05. Table 5 shows the average selected sample size obtained when the maximum value of the experiment’s p-value was > 0.05. As shown in Table 5, setting the caliper at 0.19 and using the ‘largest’ method resulted in a larger sample. In the same way, it was possible to select a caliper by evaluating the sample sizes when the first quartile of p-values from the experiment exceeded 0.05 and when the minimum p-value of the experiment exceeded 0.05. Tables 6 and 7 present the results of this process. Similarly, we can see that the ‘largest’ method with a caliper of 0.21 was the least likely method to lose samples. Therefore, we selected two candidate calipers—0.19 and 0.21—and used the ‘largest’ matching method based on the results of 100 replicated experiments.

**Fig. 5.**
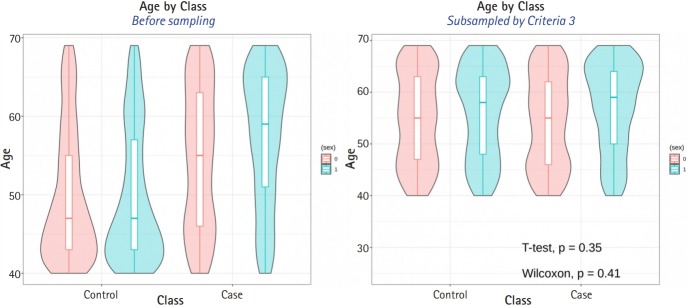
Compare age distribution between before propensity score matching (PSM) and after PSM.

**Fig. 6.**
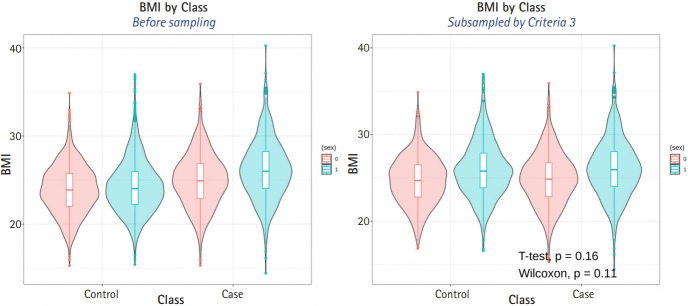
Compare body mass index (BMI) distribution between before propensity score matching (PSM) and after PSM.

The average sample sizes for nine combinations obtained using three matching methods (‘largest’, ‘smallest’, and ‘random’) and three criteria for the experiment’s p-value (minimum value, maximum value, or first-quartile value >0.05) are shown in Tables 5–7. The ‘largest’ matching method with a caliper of 0.19 (with the maximum value of the experiment’s p-value >0.05) and 0.21 (with the minimum/first-quartile value of the experiment’s p-value >0.05) resulted in a smaller loss of samples than other calipers. To guarantee the consistency of results from PSM, we set the matching method as ‘largest’ and considered both 0.19 and 0.21 as candidate calipers. The sample sizes of the training set and the test set after applying PSM with these two candidate calipers are shown in Table 4. Figs. 5 and 6 present the box plots of age and BMI before and after PSM, respectively.

### Model prediction

**Fig. 7.**
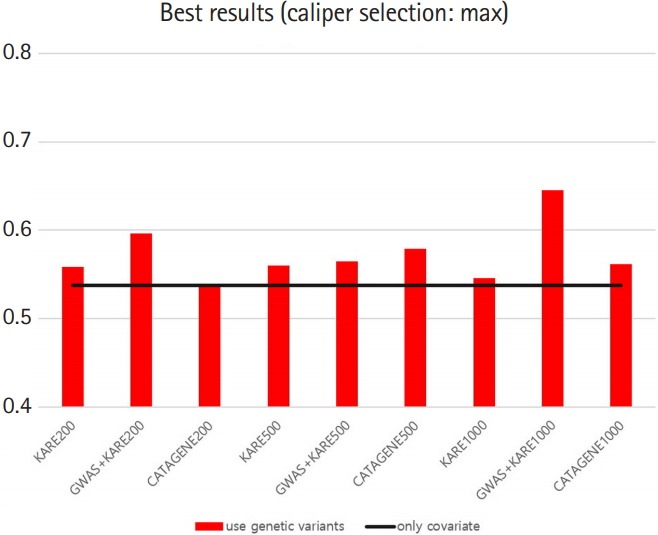
Graph of best area under the receiver operating characteristic curve results with caliper = 0.19. KARE, Korea Association Resources; GWAS, genome-wide association study

**Fig. 8.**
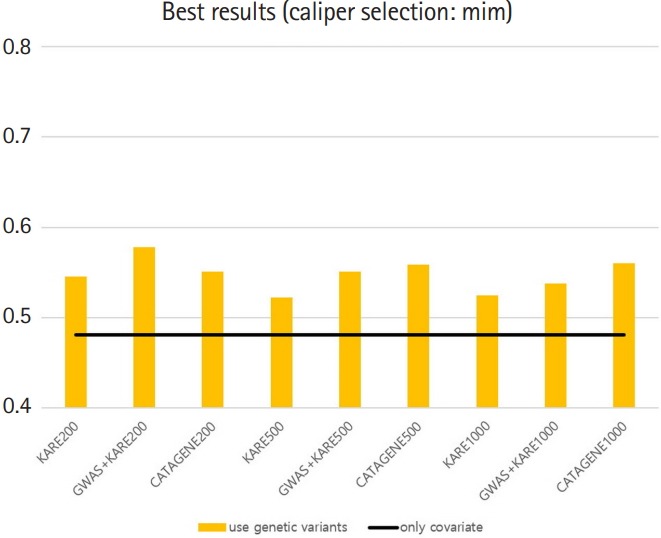
Graph of best area under the receiver operating characteristic curve results with caliper = 0.21. KARE, Korea Association Resources; GWAS, genome-wide association study

Table 8 shows the best variable selection methods, groups, and prediction models for each SNP set that we constructed. For the method without PSM, the AUC of the prediction model with both SNPs and demographic variants was close to the AUC of the model with demographic variables only (delta = –0.0029) (Table 8). However, the use of PSM with a variety of variable selection methods yielded higher AUCs for the prediction models including SNPs than for those using only demographic variables (Table 8). The best AUCs using SNPs ranged from 0.52 to 0.65. For example, group 1 in the GWAS+KARE-psmmax1000 set using the LASSO-LASSO (variable selection–prediction model) combination yielded an AUC of 0.645, which was 0.107 higher than that of the model with only demographic variables. We summarize the AUC results in Figs. 7 and 8.

## Discussion

In this study, we used multiple statistical methods (SLR, LASSO, and EN) to select variables and various SNP sets to build prediction models of T2D. Then, we compared the AUCs of the models for each SNP set. The AUCs of the models with both SNPs and demographic covariates were close to those of the models with only covariates. This result suggests that age, sex, and BMI may be good predictors of T2D in our data.

Moreover, to estimate the pure additive contribution of SNPs in our data, we applied PSM to regulate the effects of these demographic variables. When constructing models using PSM, the AUCs of models with both SNPs and covariates were higher than those of models with only covariates. For each SNP set using PSM, we constructed the best models, which had AUC values that were on average 0.051 higher than those of the corresponding models with only demographic variables. In addition, the AUC results suggest that that the prediction of T2D may be improved by up to 0.1 by adding certain SNPs.

The largest improvement obtained by adding SNPs (delta = 0.1070) was found for the model with group 1 of the GWAS + KARE-psmmax 1000 set using the LASSO-LASSO method (variable selection and prediction model). Table 9 summarizes the SNPs that were used in this model. Some of the genes in Table 9 have been identified as related to T2D by other GWASs according to the GWAS catalog. In addition, some genes were already known to be related to T2D. For example, *JAZF1, KCNJ11*, and *KCNQ1* were previously shown to be related to insulin secretion [29]. In addition, *IGF2BP2* and *CDKAL1* were reported to be associated with reduced beta-cell function [20]. Both insulin secretion and beta-cell function play important roles in T2D.

Some further studies are desirable to extend our study. First, there are multiple ways to match controls with cases. For example, Euclidian distance seems to be a promising way of matching cases and controls [30]. Second, PSM might be applied to the variable selection step by considering the pure additive contribution of genetic variants. Third, the pure additive contribution of genetic variants estimated by applying PSM may be used to estimate heritability, which needs further investigation.

## Figures and Tables

**Table 1.** Type 2 diabetes (T2D) diagnostic criteria

	T2D group	Normal subjects
Fasting plasma glucose (mg/dL)	≥ 126	≤ 100
Glycated hemoglobin (%)	≥ 6.5	< 5.7
2-Hour postprandial blood glucose (mg/dL)	≥200	≤140
History of diabetes	Treatment for T2D	No history of diabetes
	Age of disease onset ≥ 40 y	

**Table 2.** Differences between type 2 diabetes cases and controls

Variable	Case	Control	Total
No. of samples	1288	3687	4975
Sex (male/female)	671/617	1,679/2,008	2,350/2,625
Age, mean ± SD (y)	55.92 ± 8.79	49.88 ± 8.31	51.44 ± 8.85
BMI, mean ± SD (kg/m^2^)	25.54 ± 3.27	24.09 ± 2.90	24.47 ± 3.06

SD, standard deviation; BMI, body mass index.

**Table 3.** List of SNP sets

SNP sets	Caliper method	No. of total SNPs
KARE-200	-	200
GWAS + KARE-200	-	200
CATAGENE-200	-	200
KARE-500	-	500
GWAS + KARE-500	-	500
CATAGENE-500	-	500
KARE-1000	-	1,000
GWAS + KARE-1000	-	1,000
CATAGENE-1000	-	1,000
KARE-psmmax200	Maximum	200
GWAS + KARE-psmmax200	Maximum	200
CATAGENE-psmmax200	Maximum	200
KARE-psmmin200	Minimum	200
GWAS + KARE-psmmin200	Minimum	200
CATAGENE-psmmin200	Minimum	200
KARE-psmmax500	Maximum	500
GWAS + KARE-psmmax500	Maximum	500
CATAGENE-psmmax500	Maximum	500
KARE-psmmin500	Minimum	500
GWAS + KARE-psmmin500	Minimum	500
CATAGENE-psmmin500	Minimum	500
KARE-psmmax1000	Maximum	1,000
GWAS + KARE-psmmax1000	Maximum	1,000
CATAGENE-psmmax1000	Maximum	1,000
KARE-psmmin1000	Minimum	1,000
GWAS + KARE-psmmin1000	Minimum	1,000
CATAGENE-psmmin1000	Minimum	1,000

SNP, single-nucleotide polymorphism; KARE, Korea Association Resources; GWAS, genome-wide association study.

**Table 4.** Data description

	Training set (cases)	Test set (cases)
Original data	3,316 (858)	1,659 (430)
PSM data)^a^	1,626 (813)	812 (406)
PSM data^b^	1,634 (817)	816 (408)

a Propensity score matching (PSM) data: dataset using the ‘largest’ maximum method with a caliper of 0.19.

b PSM data: dataset using the ‘largest’ minimum method with a caliper of 0.21.

**Table 5.** Average sample number when the maximum value of the experiment’s p-values was >0.05

Matching method	Average selected sample number	Caliper
Largest	2,506	0.19
Smallest	2,408	0.62
Random	2,450	0.19

**Table 6.** Average sample number when the first-quartile value of the experiment’s p-values was >0.05

Matching method	Average selected sample number	Caliper
Largest	2,512	0.21
Smallest	2,439	0.75
Random	2,453	0.21

**Table 7.** Average sample number when the minimum value of the experiment’s p-values was >0.05

Matching method	Average selected sample number	Caliper
Largest	2,512	0.21
Smallest	2,458	0.82
Random	2,455	0.22

**Table 8.** Best results in each SNP set

SNP set	Method^a^ (group)	Covariates	SNPs + covariates	Delta
KARE-200	EN-LASSO (5)	0.7479	0.7451	-0.0029
GWAS + KARE-200	EN-SLR (3)	0.7479	0.7479	0
CATAGENE-200	SLR-SLR (4)	0.7479	0.7479	0
KARE-500	EN-SLR (5)	0.7479	0.7479	0
GWAS + KARE-500	EN-SLR (5)	0.7479	0.7479	0
CATAGENE-500	EN-SLR (4)	0.7479	0.7479	0
KARE-1000	EN-SLR (5)	0.7479	0.7479	0
GWAS + KARE-1000	EN-SLR (4)	0.7479	0.7479	0
CATAGENE-1000	SLR-LASSO (4)	0.7479	0.7479	0
KARE-psmmax200	LASSO-SLR (1)	0.5379	0.5585	0.0206
GWAS + KARE-psmmax200	SLR-SLR (1)	0.5379	0.5964	0.0585
CATAGENE-psmmax200	EN-LASSO (5)	0.5379	0.538	0.0001
KARE-psmmax500	LASSO-LASSO (5)	0.5379	0.5604	0.0225
GWAS + KARE-psmmax500	EN-EN (2)	0.5379	0.5645	0.0265
CATAGENE-psmmax500	EN-EN (3)	0.5379	0.5792	0.0413
KARE-psmmax1000	EN-EN (2)	0.5379	0.5461	0.0082
GWAS + KARE-psmmax1000	LASSO-LASSO (1)	0.5379	0.6449	0.107
CATAGENE-psmmax1000	LASSO-EN (3)	0.5379	0.562	0.0241
KARE-psmmin200	LASSO-EN (3)	0.4808	0.5458	0.065
GWAS + kare-psmmin200	SLR-SLR (2)	0.4808	0.5783	0.0975
CATAGENE-psmmin200	EN-EN (3)	0.4808	0.5505	0.0698
KARE-psmmin500	EN-LASSO (2)	0.4808	0.5222	0.0314
GWAS + kare-psmmin500	SLR-SLR (1)	0.4808	0.5507	0.0699
CATAGENE-psmmin500	EN-EN (2)	0.4808	0.5584	0.0777
KARE-psmmin1000	LASSO-LASSO (3)	0.4808	0.5244	0.0437
GWAS + kare-psmmin1000	EN-EN (3)	0.4808	0.5374	0.0566
CATAGENE-psmmin1000	EN-LASSO (2)	0.4808	0.5604	0.0696

SNP, single-nucleotide polymorphism; KARE, Korea Association Resources; EN, elastic-net; LASSO, least absolute shrinkage and selection operator; GWAS, genome-wide association study; SLR, stepwise logistic regression.

a Method: variable selection-prediction model.

**Table 9.** SNPs and gene locations in the GWAS + KARE psmmax top1000 LASSO-LASSO model

SNP	Gene	SNP	Gene
rs4275659	*ABCB9*	rs5215	*KCNJ11*
rs2838820	*ADARB1*	rs8181588	*KCNQ1*
rs515071	*ANK1 LOC100129400*	rs163177	*KCNQ1*
rs919115	*C10orf59*	rs4731420	*LOC100131212*
rs1048886	*C6orf57*	rs4607103	*LOC730057*
rs12924439	*CDH13*	rs6445525	*MAGI1*
rs9460546	*CDKAL1*	rs8032675	*MAP2K5*
rs7767391	*CDKAL1*	rs3761980	*MAPK14 SLC26A8*
rs2328549	*CDKAL1*	rs254271	*PRPF31*
rs10870527	*CHFR*	rs7403531	*RASGRP1*
rs12075929	*COL24A1*	rs7593730	*RBMS1*
rs17045328	*CR2*	rs10030238	*RNF150*
rs17072023	*DOCK2*	rs11855644	*SCAPER*
rs2845573	*FADS2*	rs12440511	*SCAPER*
rs1799884	*GCK*	rs560792	*SCD PRO1933*
rs780094	*GCKR*	rs9552911	*SGCG*
rs1470579	*IGF2BP2*	rs8192675	*SLC2A2*
rs864745	*JAZF1*	rs2548724	*SLCO4C1*
rs4275659	*ABCB9*	rs10933537	*TMEM16G*

SNP, single-nucleotide polymorphism.
